# *Klebsiella pneumoniae* Carbapenemase-producing Enterobacteria in Hospital, Singapore

**DOI:** 10.3201/eid1808.110893

**Published:** 2012-08

**Authors:** Indumathi Venkatachalam, Jeanette Teo, Michelle N.D. Balm, Dale A. Fisher, Roland Jureen, Raymond T.P. Lin

**Affiliations:** National University Hospital, Singapore

**Keywords:** Klebsiella pneumoniae carbapenemase, KPC-producing, Enterobacteriaceae, vancomycin-resistant enterococci, antimicrobial-resistant bacteria, Singapore, bacteria, *Suggested citation for this article*: Venkatachalam I, Teo J, Balm MND, Fisher DA, Jureen R, Lin RTP. *Klebsiella pneumoniae* carbapenamase-producing enterobacteria in hospital, Singapore, 2011 [letter]. Emerg Infect Dis [serial on the Internet]. 2012 Aug [*date cited*]. http://dx.doi.org/10.3201/eid1808.110893

**To the Editor:** During the past decade, enterobacteria that produce *Klebsiella pneumoniae* carbapenemase (KPC) have become established in the United States and countries in South America and Europe (*1*). In Asia, KPC was reported in the People’s Republic of China in 2007 (*2*) and subsequently in South Korea (*3*) and Taiwan (*4*). Public health agencies emphasize screening and strict contact precautions to control multidrug resistant *Enterobacteriaceae* (*5*). Routine testing for mechanisms of resistance facilitates detection of emerging carbapenem-resistant *Enterobacteriaceae*.

In Singapore's 1,000-bed National University Hospital during November 2010–January 2011, we identified New Delhi metallo-β-lactamase 1–producing *Enterobacteriaceae* in 2 clinical specimens but none that produced KPC (I. Venkatachalam et al., unpub. data). We conducted a laboratory screening study to determine the prevalence and nature of carbapenem-resistant *Enterobacteriacea* in April 2011. Ethics committee approval was waived for this study.

Testing of rectal swab samples is part of an established hospitalwide program for vancomycin-resistant enterococci screening. Using a scoring system to identify patients at high risk for vancomycin-resistant enterococci (*6*), we found that ≈2.5 specimens per 100 admissions were attained each month. During our study, we also tested these samples for carbapenemase-producing *Enterobacteriaceae*.

During April–June 2011, we incubated specimens for 24 h in 10 mL tryptic soy broth containing 1 mg/L imipenem, then streaked 100 µL of the broth onto CHROMagar KPC (CHROMagar, Paris, France). Colonies detected after 24 h incubation at 35°C were identified by using MALDI-TOF MS with a Microflex LT instrument (Bruker Daltonik GmbH, Leipzig, Germany). Imipenem and meropenem MICs for *Enterobacteriacaeae* were confirmed by using Etests (bioMérieux, Marcy l'Etoile, France). Isolates with MIC >2 µg/mL underwent analysis with /Metallo-β-Lactamase Confirmative Identification Pack (Rosco Diagnostica, Taastrup, Denmark) and Etest MBL (bioMérieux) for metallo-β-lactamase production. Isolates suspected to be producers were genotypically confirmed by PCR.

Of the 201 nonduplicate samples processed, 79 microorganisms exhibited imipenem resistance and were isolated on CHROMagar KPC (Table). Among *Enterobacteriaceae*, carbapenem MIC >2 μg/mL was present in 1 *E. aerogenes,* 2 *E. cloacae*, and 4 *K. pneumoniae* isolates. One isolate (*K. pneumoniae*) had a positive combined disc test result with a pattern suggestive of serine carbapenemase production.

**Table:** Bacteria isolated on CHROMagar in screening for carbapenemase-producing *Enterobacteriaceae*, Singapore, 2011*

Organism	No. isolated
*Enterobacteriaceae*	
* Klebsiella pneumoniae*	11
* Enterobacter cloacae*	3
* Enterobacter aerogenes*	2
* Proteus mirabilis*	2
* Escherichia coli*	1
* Serratia marcescens*	1
Non-*Enterobacteriaceae*: gram-negative nonfermenters	
* Pseudomonas aeruginosa*	20
* Acinetobacter baumannii*	17
* Stenotrophomonas maltophilia*	13
* Elizabethkingia meningoseptica*	3
* Wautersiella falsenii*	1
*Enterococci*	
* Enterococcus gallinarum*	4
* Enterococcus faecalis*	1

*CHROMagar, Paris, France.

We analyzed genomic DNA (DNeasy Blood and Tissue Kit, QIAGEN, Hilden, Germany) from this isolate by using PCR for transmissible carbapenem resistance markers: metallo-β-lactamases (VIM, IMP, and KHM-1), serine carbapenemases (KPC, GES1–5 and 7), and OXA-48. *bla*_KPC_-specific primers (forward primer 5′-CGTTGACGCCCAATCC-3′; reverse primer 5′-ACCGCTGGCAGCTGG-3′) generated a 390-bp amplicon. Full gene sequencing of *bla*_KPC_ (forward primer 5′-ATGTCACTGTATCGCCGTCT-3′; reverse primer 5′-CCTAAATGTGACAGTGGTTGG) revealed 100% homology to *bla*_KPC-2_ (GenBank accession no. FJ628167.2). Further analysis showed that the isolate carried extended-spectrum β-lactamase (*bla*_TEM-1_, *bla*_SHV-11_, *bla*_CTX-M-15_), plasmid-located AmpC (*bla*_DHA-1_), and 16S rRNA methylase *armA* genes but was negative for *bla*_CMY_, *bla*_OXA_, *bla*_GES_, metallo-β-lactamases, and plasmid-mediated quinolone (*qnr*) genes. Multilocus sequence typing conducted at Institut Pasteur (Paris, France), identified this isolate as sequence type 11. It was susceptible only to colistin and tigecycline.

Sequence type 11, a single-locus variant of the internationally dominant sequence type 258 clone (*7*), is present in 64.2% of KPC-producing *K. pneumoniae* in China (*8*). In South Korea, sequence type 11 is the most common clone of extended-spectrum β-lactamase–producing *K. pneumoniae* isolates (*3*).

The KPC-producing *K. pneumoniae* originated from a woman in the local community, 89 years of age, who had severe ischemic cardiomyopathy and atrial fibrillation. She was discharged home after a 3-day hospitalization for treatment of stroke in January 2011. During May 2011, she was readmitted after a severe stroke. During week 4, she was transferred to a subacute care hospital but readmitted within 24 hours with a lower respiratory tract infection. A rectal swab sample was collected for routine screening for vancomycin-resistant enterococci. We empirically prescribed a 10-day course of piperacillin-tazobactam. On day 10 of treatment, KPC-producing *K. pneumoniae* was isolated from the rectal specimen. The patient responded to treatment and was discharged to a long-term care facility.

This case demonstrates concerns about a KPC of local community origin because no other KPC-producing *Enterobacteriaceae* were isolated during this inpatient surveillance and the patient had neither received antimicrobial drugs nor traveled in the 6 months before her May admission (*7*). However, she was admitted 3 weeks before sampling; an unidentified hospital source remains a possibility. Of added concern is the potential for dissemination within the facility to which she was discharged.

Resistance to third-generation cephalosporins was reported for 20% of *Escherichia coli*, 32.3% of *K. pneumoniae*, 46.2% of *Acinetobacter* spp., and 7.5% of *Pseudomonas aeruginosa* clinical isolates at 4 major Singapore hospitals during January 2006–December 2008 (*9*). Authors reported positive correlation between meropenem administration and carbapenem resistance development in *Acinetobacter* spp. blood isolates.

When the resistance mechanism to an antimicrobial drug is embedded in highly mobile elements like plasmids, widespread dissemination is possible. Although acute care hospitals are conducive to development of antimicrobial drug resistance, long-term care facilities facilitate spread of these organisms (*10*). Infection control interventions including routine screening for mechanisms of resistance and responsible use of antimicrobial drugs are increasingly critical in hospitals and long-term care facilities; a response plan coordinated between these facilities is needed.
